# Polystyrene-bound AlCl_3_ – a catalyst for the solvent-free synthesis of aryl-substituted tetrazoles†

**DOI:** 10.1039/d4cy01215a

**Published:** 2025-02-07

**Authors:** Max Schmallegger, Mathias Wiech, Sebastian Soritz, Miriam de J. Velásquez-Hernández, Brigitte Bitschnau, Heidrun Gruber-Woelfler, Georg Gescheidt

**Affiliations:** a Institute of Physical and Theoretical Chemistry, Graz University of Technology Stremayrgasse 9 8010 Graz Austria schmallegger@tugraz.at g.gescheidt-demner@tugraz.at; b Institute of Process and Particle Engineering, Graz University of Technology Inffeldgasse 13 8010 Graz Austria

## Abstract

Tetrazole moieties are components of various pharmacologically active molecules. Several synthetic protocols for the synthesis of tetrazoles have been developed. Among those, the reaction of organic nitriles with azides catalyzed by Lewis acids (LAs) provides a convenient access. Nevertheless, generally rather harsh reaction conditions have to be utilized for such syntheses. We have developed a simple, solvent-free procedure which allows a convenient isolation of tetrazoles using a heterogeneous catalyst: we show that polystyrene/AlCl_3_ composites produce tetrazoles at reasonable yields and allow a simple work-up procedure. We have characterized the AlCl_3_/polystyrene composite (gas sorption, XRD, IR) and investigated its efficacy in the preparation of aryl-substituted tetrazoles. We also have evaluated MgCl_2_, CuCl_2_, and ZnCl_2_ as Lewis-acid catalysts, but they are clearly outperformed by AlCl_3_ correlating with the Lewis-acid strength on the Gutmann-Beckett scale.

## Abstract 2

We present photopolymerized polystyrene as a support for AlCl3, which is used for the synthesis of tetrazoles under solvent-free conditions.

## Graphical abstract

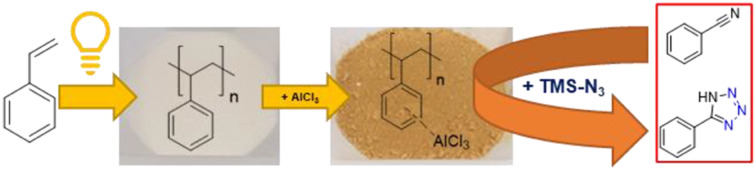

## Introduction

Tetrazole-containing compounds have been used in a wide range of applications: they are employed as ligands, in photography, as precursors for more complex heterocycles and in drug development.^1–8^ Several tetrazole-containing drugs with a variety of biological activities are available on the market, including antihypertensive, antimicrobial, antiviral, antiallergic, cytostatic, and nootropic functions.^3,7,9^ 5-monosubstituted tetrazoles in particular have been widely used in pharmaceutics because they can serve as a bio-isosteric replacement for carboxylic acids, exhibiting similar biological activity and improved resistance to metabolic degradation.^10,11^

A widely used approach for the synthesis of 5-monosubstituted tetrazoles is the diazotization of imidohydrazides.^12^ Alternatively, they can be synthesized from amines,^13,14^ amides,^15^ and aldoximes,^16,17^ or in multi-component reactions, such as the Ugi tetrazole^6,18^ reaction or the Passerini tetrazole reaction.^19^ However, the most commonly used synthetic route involves the reaction of inorganic azide salts or organic azides with nitriles.^8,20,21^ This approach was first introduced in 1958, utilizing sodium azide and ammonium chloride in dimethylformamide.^22^ The use of Lewis-acid (LA) catalysis with organometallic or organosilicon azides has been shown to be efficient.^11,23,24^ Sharpless and co-workers^25–27^ presented systematic studies, showing that the activation of the nitrile by an electron-withdrawing group is a prerequisite for an efficient reaction.^26,28,29^

While the use of Lewis acids as catalysts is a well-established approach, it still suffers from certain shortcomings. This includes low reaction yields, harsh reaction conditions, the use of expensive catalysts, time-consuming work-up procedures, or the need for high-boiling point solvents.^12,30–33^ Therefore, the development of more efficient and convenient methods for the synthesis of tetrazoles is desired.^8,17,34,35^

In this study, we evaluated AlCl_3_, MgCl_2_, CuCl_2_, and ZnCl_2_ as Lewis acid catalysts for the synthesis of 5-monosubstituted tetrazoles *via* a solvent-free reaction. We correlated their Lewis acid strength, as determined by the Gutmann–Beckett method, with tetrazole product yields. AlCl_3_ was found to be the most efficient catalyst under our experimental conditions. It has already been shown that immobilized AlCl_3_ (*e.g.*, on polymers) is a well-suited heterogeneous catalyst.^36–42^ Based on the early work by Neckers *et al.*,^43,44^ we report on the feasibility of using AlCl_3_ supported on a custom-made porous polystyrene photopolymer^45^ matrix for the solvent-free synthesis of 5-aryl tetrazoles from trimethylsilyl azide and arylnitrile derivatives. The prepared composites are characterized and tested on nitriles presenting substituents with electron donating and -withdrawing abilities.

## Experimental section

### Materials

Nitriles (R–N_3_), aluminium chloride (AlCl_3_), and styrene were purchased from Sigma-Aldrich. Trimethyl silyl azide (TMS–N_3_) and divinylbenzene were purchased from TCI Chemicals. Zinc chloride (ZnCl_2_), copper chloride (CuCl_2_), magnesium chloride (MgCl_2_) and isopropanol (i-PrOH) were purchased from Roth. All substances were acquired in the highest purity available and used without further purification.

### Preparation of polymer-bound AlCl_3_

Porous polystyrene was prepared following the procedure described by Seeberger *et al.*^45^ 2.8 ml styrene, 2.2 ml divinyl benzene, 5 ml methanol and 170 mg of the commercial photoinitiator “Irgacure 819” (bis(2,4,6-trimethylbenzoyl)-phenylphosphine oxide) were mixed in a 10 ml glass vial. The mixture was placed in an Anycubic Wash & Cure 2.0 device (*λ* = 405 nm) and irradiated for 60 minutes. The obtained monolith was washed with ethanol and dried at 80 °C for 2 hours. 500 mg of the polymer was then ground and placed in a round-bottomed flask. 1000 mg AlCl_3_ and 20 ml of CCl_4_ were added to the mixture.^46^ The polymer/AlCl_3_ ratio was rationalized by testing composites with different mass ratios (polymer : AlCl_3_ = 1 : 1 and 1 : 3, respectively), which show inferior catalytic activity (see Fig. S14†). The mixture was heated to 50 °C and stirred for one hour until a brown solid was obtained. The composite was filtered, washed three times with deionized water and ethanol, and dried at 120 °C for two hours.

### Characterization of the polymer-bound AlCl_3_ catalyst

#### Infrared spectroscopy

FT-IR spectra were recorded on a Bruker Alpha spectrometer in reflection mode using OPUS 7.5 software.

#### Gas sorption analysis

N_2_ adsorption isotherms of each sample were recorded at 77 K on a Micromeritics 3FLEX instrument using He for free-space determination. Prior to the measurements, the powdered material was degassed at 393 K for 12 h under vacuum. The specific surface area was calculated by employing the Brunauer–Emmett–Teller (BET) method using the consistency criteria suggested in the literature.^47,48^ The BET equation was applied to the experimental N_2_ isotherm within a relative pressure range of *P*/*P*_0_ = 0.08–0.35. The pore size distribution was obtained using density functional theory (NLDFT).

#### X-ray powder diffraction

Powder X-ray diffraction (PXRD) analysis was performed using an XRDynamic 500 diffractometer (Anton Paar, Graz, Austria) with Bragg Brentano geometry in the 2*θ* range of 5° to 110° with a step size of 0.02° and a Cu-K_α_ X-ray source (*λ* = 1.5418 Å). Data evaluation was performed using the X'Pert Highscore Plus software.

### Synthesis of tetrazoles

#### Lewis acids as catalysts

Solvent-free reactions using Lewis acids as heterogeneous catalysts were performed employing 9.8 μL of trimethylsilyl azide and 5 μL of benzonitrile (1.5 eq. molar excess of azide) and 0.05 mmol (= 10 mol%) of the corresponding metal salt. The mixture was then heated to 160 °C for 16 h. Subsequently, 800 μL of DMSO-*d*_6_ and 20 μL of 33% HCl-*d*_1_ in H_2_O-*d*_2_ were added. After centrifugation at 16 000 rpm for 6 min, the supernatant was transferred to an NMR tube and measured directly.

#### Polymer-bound AlCl_3_ as heterogeneous catalysts

The reactions using polymer-bound AlCl_3_ as a heterogeneous catalyst were performed using 50 μL of trimethylsilyl azide, 25 mg of the corresponding nitrile, and 20 mg (corresponding to 5 mol% of Al) of the catalyst. The mixture was then heated to 160 °C for 6 h. Subsequently, 800 μL of DMSO-*d*_6_ and 20 μL of 33% HCl-*d*_1_ in H_2_O-*d*_2_ were added. The polymer was removed by filtration, and the filtrate was directly analyzed by NMR. For subsequent reaction runs, polymer-bound AlCl_3_ was recycled by washing once with DMSO and then with ethanol, and dried at 80 °C for 2 h. Subsequently, the catalysts were reused according to the procedure described above. For leaching tests, “hot filtration” was employed. This refers to the removal of catalyst during the reaction. Here, it was assumed that the catalyst leached from the polymer support maintained some activity in the mixture. Samples were prepared as previously described. After 1 hour, the polymer-bound AlCl_3_ was removed from the reaction by filtration. The samples were further kept at 160 °C for the corresponding reaction time (2 h, 4 h or 6 h respectively). The reaction yield was determined as described below.

### Determination of reaction yield by ^1^H NMR spectroscopy

^1^H NMR spectra were recorded on a 200 MHz Bruker Avance DPX spectrometer. Chemical shifts (*δ*) are reported in ppm relative to tetramethylsilane (TMS), using the residual non-deuterated solvent as an internal reference. The reaction yield was determined by comparing the integrals of characteristic signals of the product and educt (see ESI†).

### Determination of Lewis acid strength by ^31^P NMR

^31^P NMR spectra were recorded on a 200 MHz Bruker Avance DPX spectrometer. Chemical shifts (*δ*) are reported in ppm, relative to 85% H_3_PO_4_ in H_2_O. The experiments were conducted in MeOH-*d*_4_.

## Results and discussion

### Lewis acid strength and catalytic performance

**Scheme 1:**
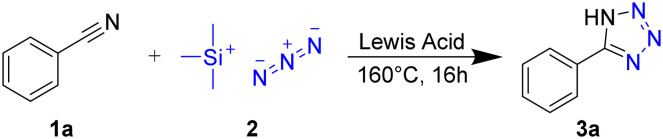
Synthesis of 5-phenyltetrazole (3a) from benzonitrile (1a) and TMS-N_3_ (2) in the presence of Lewis acids at elevated temperatures.

As a benchmark to screen the catalytic activity of Lewis acids in the synthesis of 5-aryltetrazoles, we reacted benzonitrile with trimethylsilyl azide in the presence of the readily available metal chlorides MgCl_2_, CuCl_2_, ZnCl_2_, and AlCl_3_ under solvent-free conditions, yielding 5-phenyltetrazole 3a (Scheme 1).

**Fig. 1:**
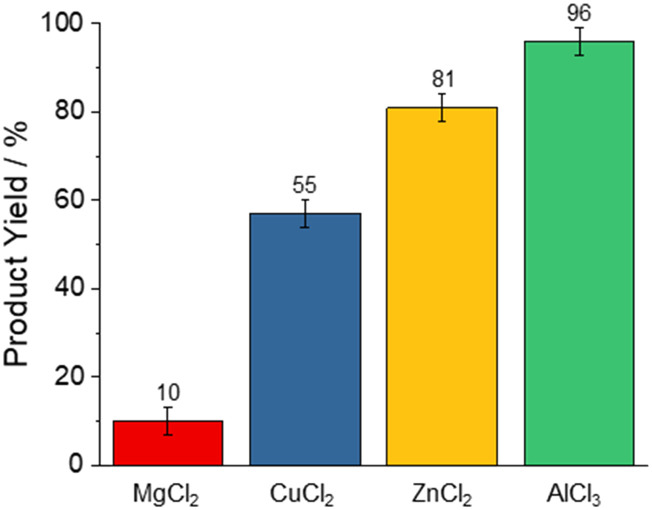
Yields of 5-phenyltetrazole with different Lewis acid catalysts at 160 °C for 16 h, as determined by ^1^H-NMR spectroscopy.

**Scheme 2:**
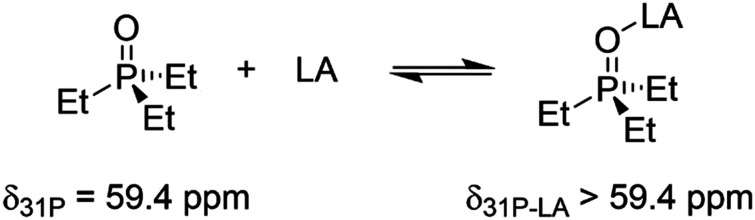
Interaction of triethylphosphine oxide with a Lewis acid, resulting in a change in chemical shift in the ^31^P NMR spectrum.

The yield of 3a was the lowest for MgCl_2_ (10%), CuCl_2_ (55%), and ZnCl_2_ (81%), peaking with AlCl_3_ (96%, Fig. 1). These reactivities correlate with the Lewis-acid strength as determined by the NMR-based Gutmann–Beckett method^49,50^ for Lewis acidity within the limits of this approach:^51^ Here, the ^31^P resonance of triethylphosphine oxide shifts downfield as a result of de-shielding from coordination with Lewis acids. This effect correlates with the strength of the electron acceptor, *i.e.* the Lewis Acid. Accordingly, stronger Lewis acids give rise to a larger chemical shift perturbation Δ*δ*^31^P (Scheme 2).^51–53^

The ^31^P chemical shifts of triethylphosphine in the presence of Lewis acids are presented in Table 1 (see ESI† for the corresponding spectra). Lewis acids that induce a higher chemical shift in the ^31^P NMR also lead to higher product formation when employed as catalysts. Based on these observations, we exclusively employed AlCl_3_ for further experiments.

**Table 1:** ^31^P chemical shifts *δ*_31P_ observed for triethylphosphine oxide (TEPO) in the presence of Lewis acids. The relative change Δ*δ*_31P_ is reported against TEPO in absence of Lewis acid. The given yields correspond to the reaction outlined in Scheme 1

Lewis acid	*δ*_31P_/ppm	Δ*δ*_31P_/ppm	3a yield/%
MgCl_2_	59.5	0.1	10
CuCl_2_	62.7	3.3	55
ZnCl_2_	64.3	4.9	81
AlCl_3_	74.3	14.9	96

### Preparation, performance and characterization of the heterogeneous catalyst

**Scheme 3:**
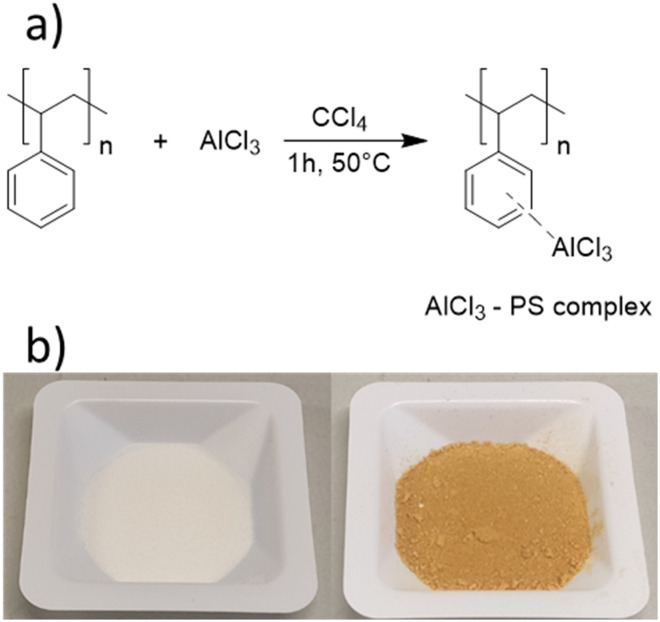
a) Reaction scheme for the immobilization of AlCl_3_ in a porous polystyrene matrix *via* crosslinking. b) Photograph of the porous polystyrene before (left) and after (right) immobilization of AlCl_3_.

To produce a porous polymer support for AlCl_3_, we employed a straightforward photochemical pathway:^45^ a mixture of styrene, the cross-linker divinyl benzene, methanol, and the photoinitiator Irgacure 819 was prepared and irradiated for 60 min with 405 nm LEDs. The resulting polymer monolith was cleaned, dried, and ground into fine powder. In the second step, an AlCl_3_/CCl_4_ solution was added to the polymer (Scheme 3a, see Experimental section for details), yielding AlCl_3_@polystyrene (Scheme 3).^54–56^ The immobilization is distinguishable by a change in colour. The parent polystyrene is white, whereas the polystyrene–AlCl_3_ composite exhibits a yellow-brownish colour.^54–56^

**Fig. 2:**
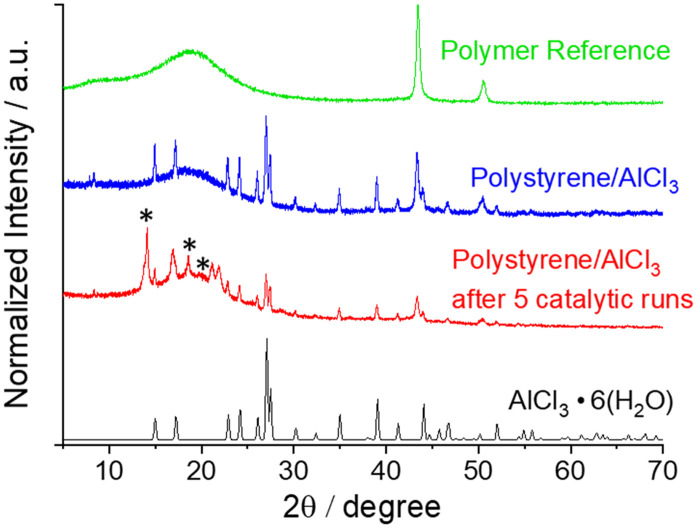
Normalized PXRD patterns of the polymer reference (green), the polystyrene/AlCl_3_ composite before (blue) and after 5 catalytic runs (red); signals stemming from hydrolysis products AlCl(OH)_2_·2(H_2_O) and Al(OH)_3_ are marked with an asterisk. The diffractogram of AlCl_3_·6(H_2_O) (ICSD-22071) is shown for comparison (black).

IR spectroscopy revealed new vibrational bands in the sample containing AlCl_3_. A strong band at approximately 1600 cm^−1^, characteristic of AlCl_3_, was detected. The broad band around 3300 cm^−1^ was attributed to hydroxyl groups from partially hydrolysed AlCl_3_. The corresponding spectra are shown in the ESI† (Fig. S9). Furthermore, X-ray powder diffraction (PXRD) measurements were performed. The AlCl_3_@polystyrene composite exhibited new reflexes compared to the blank polymer reference (Fig. 2). These are attributable to AlCl_3_·6(H_2_O) based on data from the ICSD database (ICSD-22071). This confirms, that upon immobilization and work-up, a substantial portion of intact AlCl_3_ is present in the composite. We also recorded PXRD patterns of the composite after employing it as heterogenous catalyst. Additional reflexes are detected after five consecutive reaction runs at 160 °C for 6 h (see below). The corresponding peaks reflect the formation of the hydrolysis products AlCl(OH)_2_·2(H_2_O) and Al(OH)_3_ due to the (partial) hydrolysis of immobilized AlCl_3_ (see Fig. S13†).^57^ This is indicative of a loss in the catalytic activity of the composite upon reuse.

For reactants to access the catalytically active species efficiently, it is critical that the composite provides a sufficiently high surface area. BET measurements revealed that the AlCl_3_–polymer composite is mesoporous, with a surface area of approximately 6 m^2^ g^−1^ (see Fig. S10†). The analysis further indicates, that the mesopores have an average pore size of 32 Å.

**Fig. 3:**
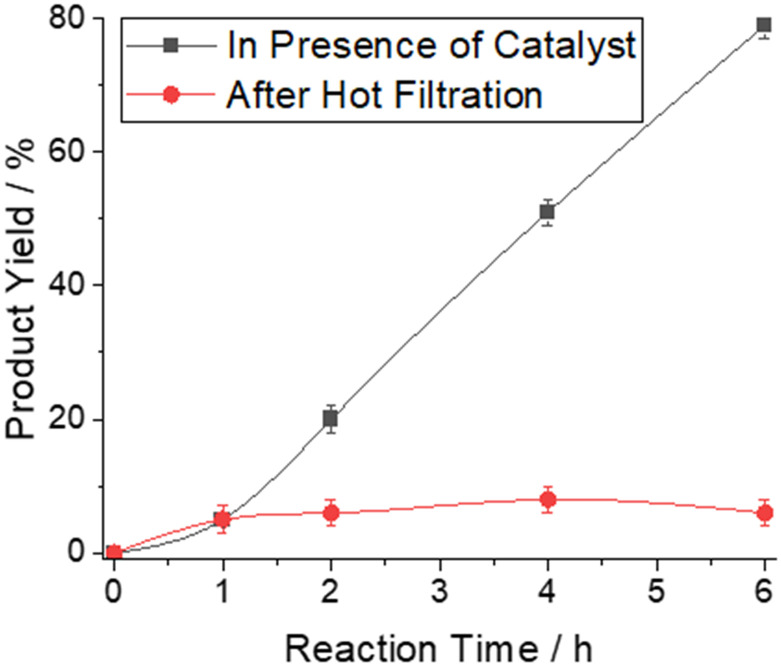
Product yield of 5-phenyltetrazole as determined by ^1^H-NMR in presence of polystyrene-bound AlCl_3_ as heterogeneous catalyst (black) or after hot filtration after 1 hour (red).

Using the AlCl_3_-containing polymer composite as a catalyst for the conversion of benzonitrile (1a) and trimethylsilyl azide (2) to the corresponding tetrazole 3a, yields of up to 79% were achieved (solvent-free, Scheme 1, 160 °C, 6 h; see Experimental section for details). We assessed the influence of reaction time on the NMR yield of 3a by stopping the reactions after 1 to 6 h (Fig. 3).

Longer reaction times did not significantly improve product yield. While yields obtained with the composite catalysts are generally lower than those obtained when employing AlCl_3_ directly (79% *vs.* 96%), the required time decreases considerably (6 h *vs.* 16 h). Furthermore, when comparing turnover number (TON) and turnover frequency (TOF), we observe almost identical values when employing the composite compared to AlCl_3_: for the conversion of 1a and 2 to the corresponding tetrazole 3a, a TON of 8.8 (compared to 9.6 for AlCl_3_) and a TOF of 1.46 h^−1^ (compared to 1.6 h^−1^ for AlCl_3_) is obtained. While higher TONs and TOFs for the catalytic tetrazole synthesis were reported^58–60^ the results still underpins the practicality of utilizing low-cost precursors along with a simple loading of AlCl_3_ leading to catalytically active composites.

Importantly, the work-up procedure becomes much more convenient: The AlCl_3_–polymer composite can be easily separated from the reaction product by filtration. However, filtration cannot remove Al species leached from the composites. To study the leaching of the catalyst, “hot filtration” tests were performed: the composite material with polymer-bound AlCl_3_ was filtered from the reaction mixture after 1 h. The samples were maintained at 160 °C for 1–5 h. For samples containing polymer-bound AlCl_3_, the product yield continued to increase with increasing time. In contrast, the yield remained constant for the sample in which the composite catalyst was removed *via* filtration (Fig. 3). Although the leaching of catalytically active Al species from the polymer cannot be entirely excluded, if it occurs, it is promptly transformed into a non-catalytically active species. This indicates that the polymer-bound AlCl_3_ truly acts as a heterogeneous catalyst.

**Fig. 4:**
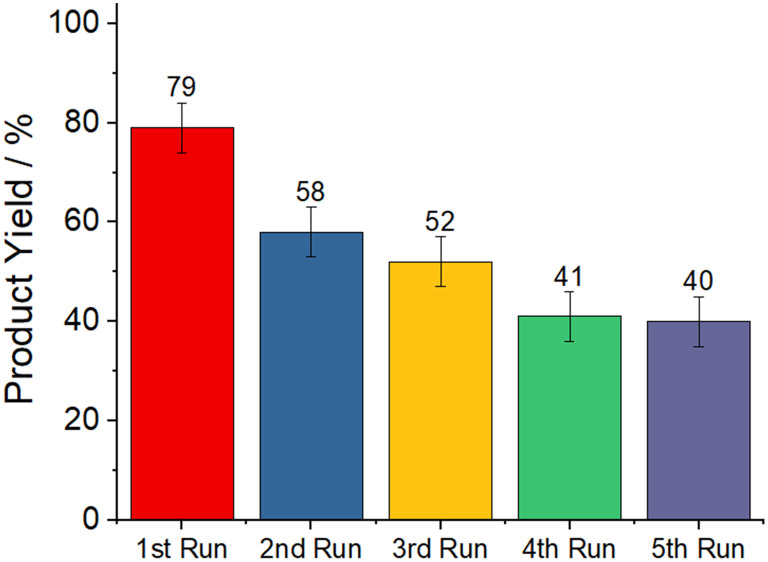
^1^H-NMR determined yields of 5-phenyltetrazole with (reused) polystyrene-bound AlCl_3_ as heterogeneous catalyst. Conditions: 160 °C for 6 hours, solvent free.

Because the polymer composite can be easily separated from the reaction mixture, we tested the activity of the catalyst upon reuse. The composite was collected, washed, dried, and reused in subsequent reaction runs. For substrate 1a, a second run yielded 58% 3a, which gradually decreased to 40% in the fifth run (Fig. 4). As indicated by the PXRD results, this inactivation is a consequence of the hydrolysis of AlCl_3_ within the composite. Leaching of catalytically active species may also contribute to the loss of reactivity.

In addition to probing the persistence of the active species, the stability of the polymer support under experimental conditions was assessed. The NMR spectra recorded from the solutions extracted between the reaction runs showed no solubilized polymer components (see Fig. S3†), illustrating the stability of the support material.

**Scheme 4:**
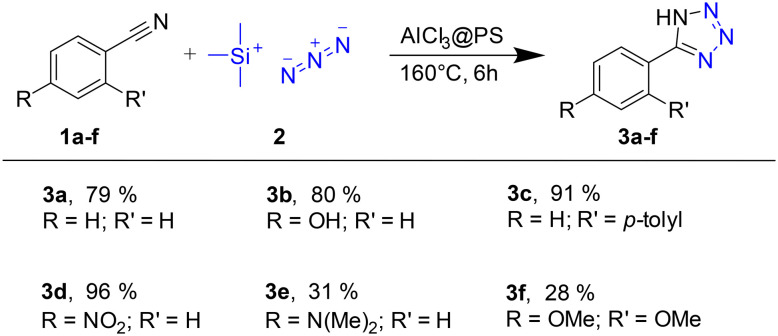
Scope of the solvent-free tetrazole synthesis; yields were determined by ^1^H-NMR.

To explore the versatility of our approach, we used nitriles 1b–f as additional starting compounds. These derivatives were chosen to represent steric (3c), electron-withdrawing (3d), and -donating (3b, 3e, 3f) effects (Scheme 4).

Except for 3e and 3f, which carry electron-donating substituents (4-dimethylamino or 2,4-dimethoxy groups, respectively), the yields of 3a–3d range from 79% to 96%. This is in line with less efficient conversions reported for analogous reactions showing that substrates with electron-withdrawing or weak electron donating groups provide higher reactivity.^26,28,29^

## Conclusions

The embedding of AlCl_3_ into porous polystyrene results in a catalyst capable of producing 5-aryl tetrazoles in yields exceeding 80%. However, when the aromatic moiety contains electron-donating substituents, yields decrease to approximately 30%. The work-up process is straightforward, as the product can be extracted by washing the AlCl_3_/polystyrene composite with DMSO. Notably, this composite offers significantly shorter reaction times (6 hours compared to 16 hours). Although AlCl_3_ is partially hydrolysed during the reaction, we did not observe any leaching of catalytically active species, as confirmed by hot filtration experiments.

Our study demonstrates that utilizing low-cost precursors along with a simple loading of AlCl_3_ leads to catalytically active composites. These composites not only provide efficient reaction times and good yields but also facilitate easy product isolation. Additionally, the use of DMSO as a solvent, instead of problematic high-boiling alternatives, underpins the practicality of this method.

## Data availability

The data supporting this article have been included as part of the ESI† See DOI: https://doi.org/10.1039/D4CY01215A.

## Conflicts of interest

There are no conflicts to declare.

## Supplementary Material

CY-015-D4CY01215A-s001
