# Supplementary material for: Polystyrene-bound AlCl3 – a catalyst for the solvent-free synthesis of aryl-substituted tetrazoles
Source: Catal Sci Technol. 2025 Feb 7;15(6):1983–8. doi: 10.1039/d4cy01215a (PMC11815551; doi:10.1039/d4cy01215a)
Supplement: CY-015-D4CY01215A-s001 [file CY-015-D4CY01215A-s001.pdf]

Supplementary Information (SI) for Catalysis Science & Technology.
This journal is © The Royal Society of Chemistry 2025

Supporting Information

Polystyrene-Bound $AlCl_3$ - a Catalyst for the Solvent-Free Synthesis of Aryl-Substituted Tetrazoles

Max Schmallegger,*[a] Mathias Wiech,[a] Sebastian Soritz,[b] Miriam de J. Velásquez-Hernández, Brigitte Bitschnau,[a] Heidrun Gruber-Woelfler[b] and Georg Gescheidt,*[a]

Contents

Determination of the Lewis Acid Strength

^{1}H NMR Spectra

ATR-IR Spectra

Gas Sorption Measurements

X-Ray Powder Diffraction Patterns

Optimization of $AlCl_3$ concentration

Determination of the Lewis Acid Strength

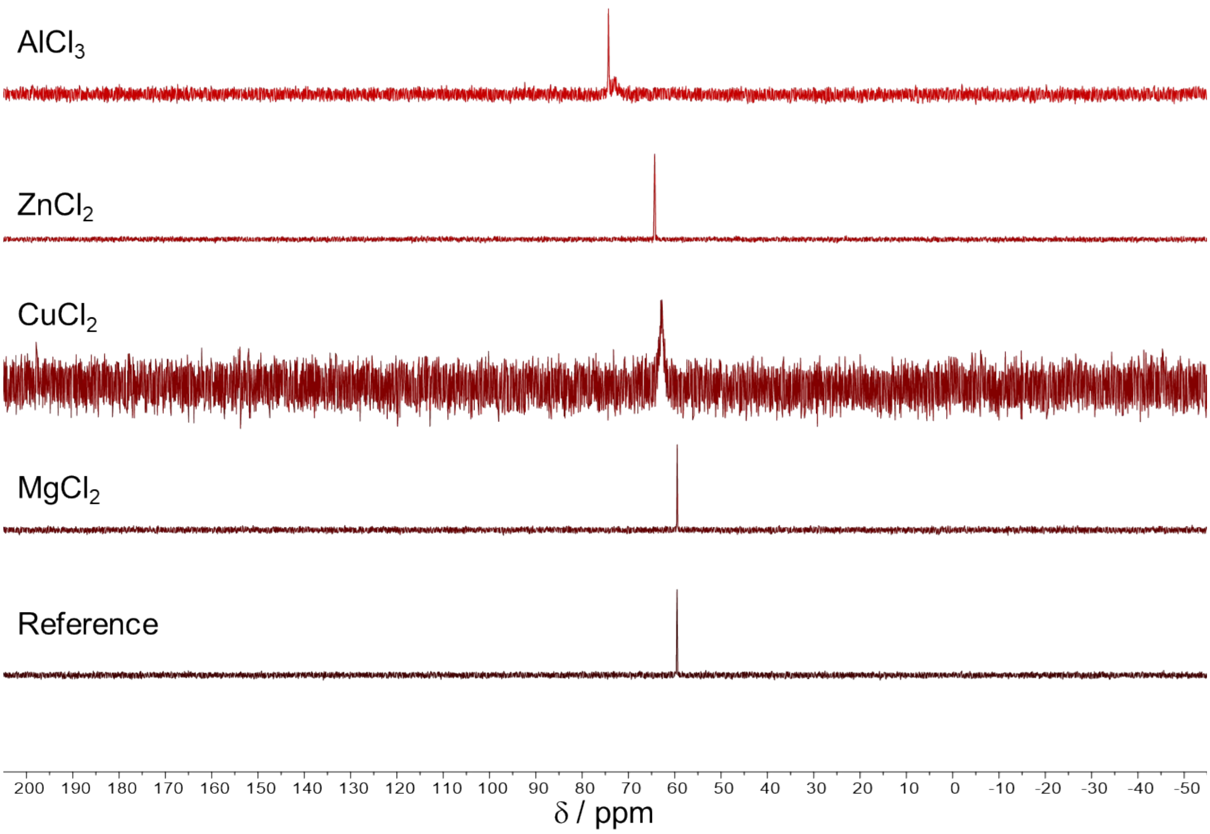

Figure S1. ^{31}P NMR spectra of TEPO in the presence of different LAs; spectra were recorded in MeOH-d_4.

The Lewis acid strength, as determined by the Gutmann-Beckett method, shows a clear correlation with the catalytic activity in the formation of tetrazoles. Lewis acids inducing a higher chemical shift in the ^{31}P NMR also lead to higher product formation when employed as catalysts.

^1H NMR Spectra

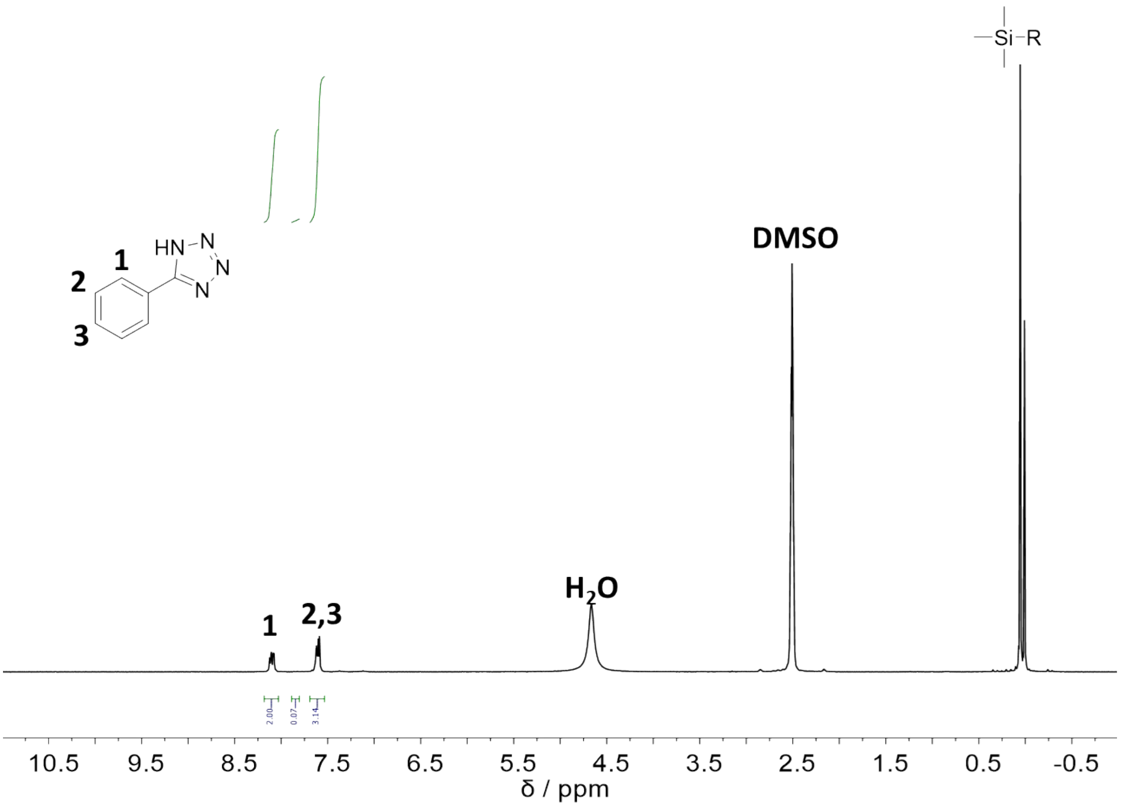

Figure S2 ^1H-NMR-spectrum of 5-phenyltetrazole formation reaction using $AlCl_3$ as catalyst in under bulk conditions at 160°C; spectrum was recorded in DMSO-d_6.

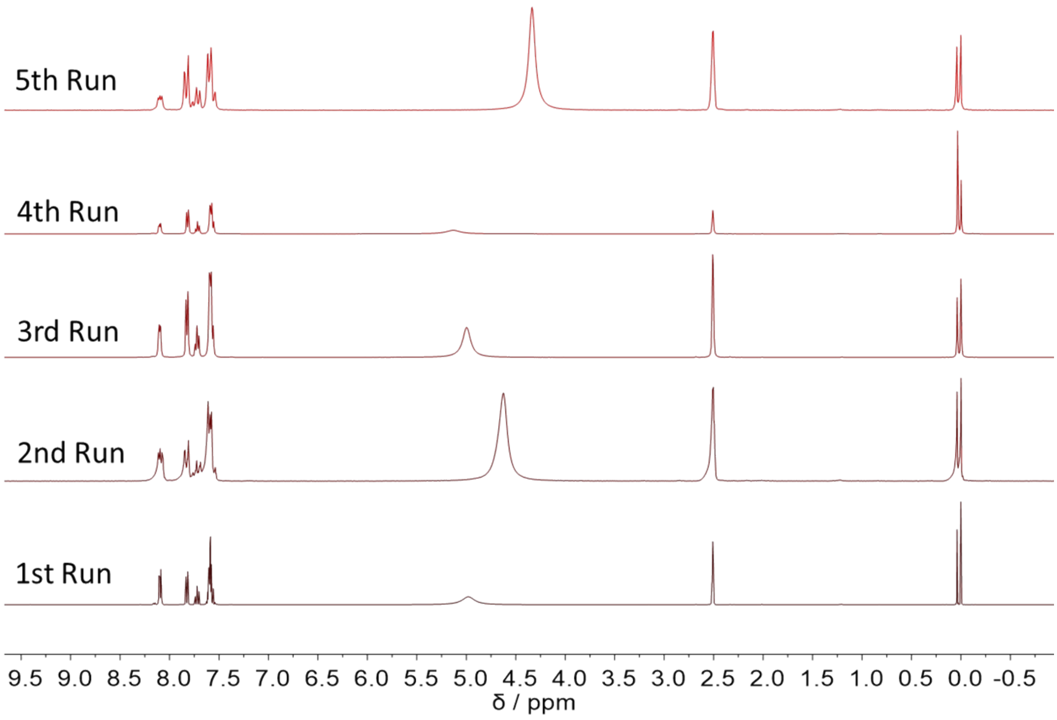

Figure S3 ^1H-NMR-spectra of the formation of 5-phenyltetrazole tetrazole using Polymer-bound $AlCl_3$ as catalyst under bulk conditions at 160°C; for all runs, the catalyst was recycled and used again; spectra were recorded in DMSO-d_6.

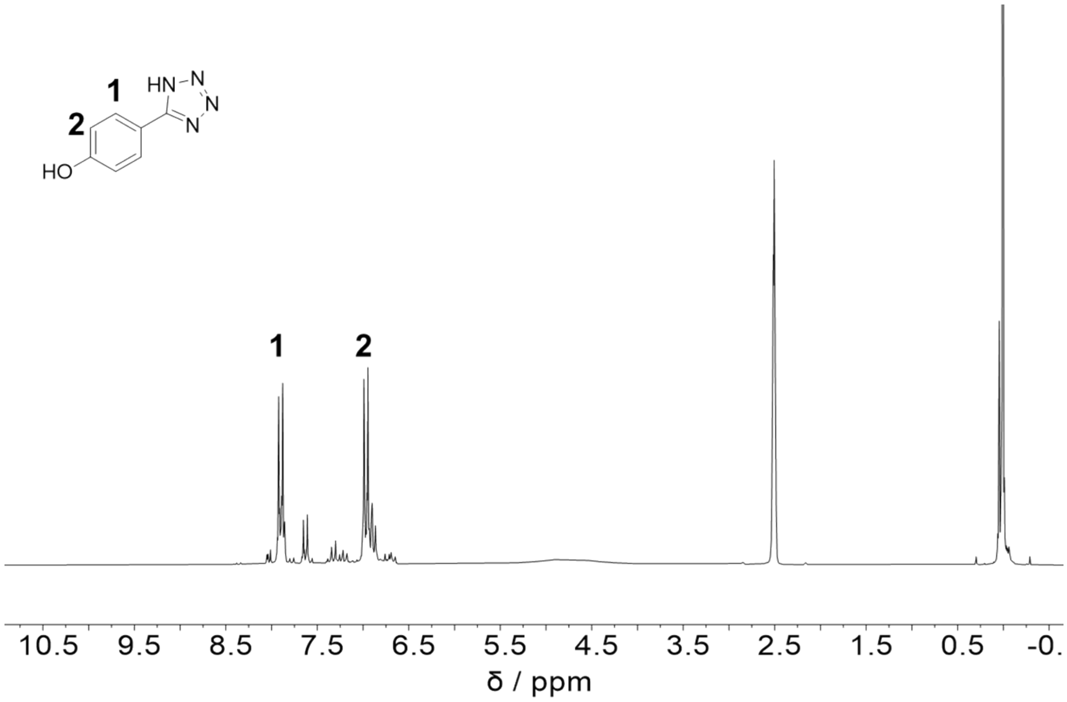

Figure S4 ^{1}H-NMR-spectrum of **3b** using polymer-bound $AlCl_3$ as catalyst in under bulk conditions at 160°C; spectrum was recorded in DMSO-d_6.

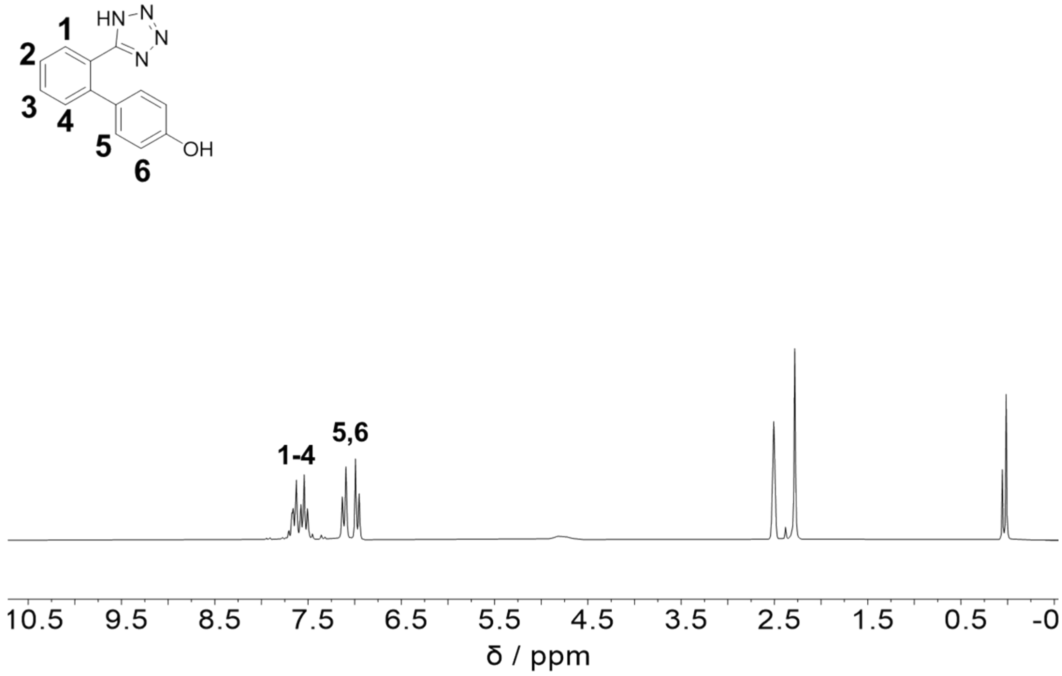

Figure S5 ^{1}H-NMR-spectrum of **3c** using polymer-bound $AlCl_3$ as catalyst in under bulk conditions at 160°C; spectrum was recorded in DMSO-d_6.

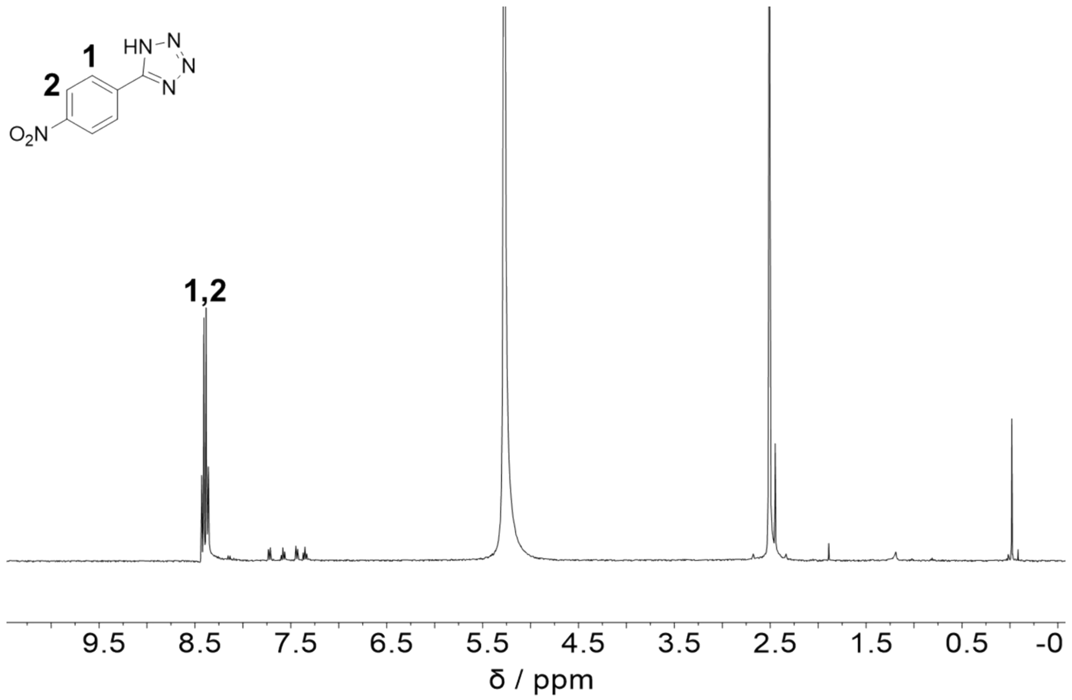

Figure S6 ^{1}H-NMR-spectrum of **3d** using polymer-bound $AlCl_3$ as catalyst in under bulk conditions at 160°C; spectrum was recorded in DMSO-d_6.

Figure S7 ^{1}H-NMR-spectrum of **3e** using polymer-bound $AlCl_3$ as catalyst in under bulk conditions at 160°C; spectrum was recorded in DMSO-d_6

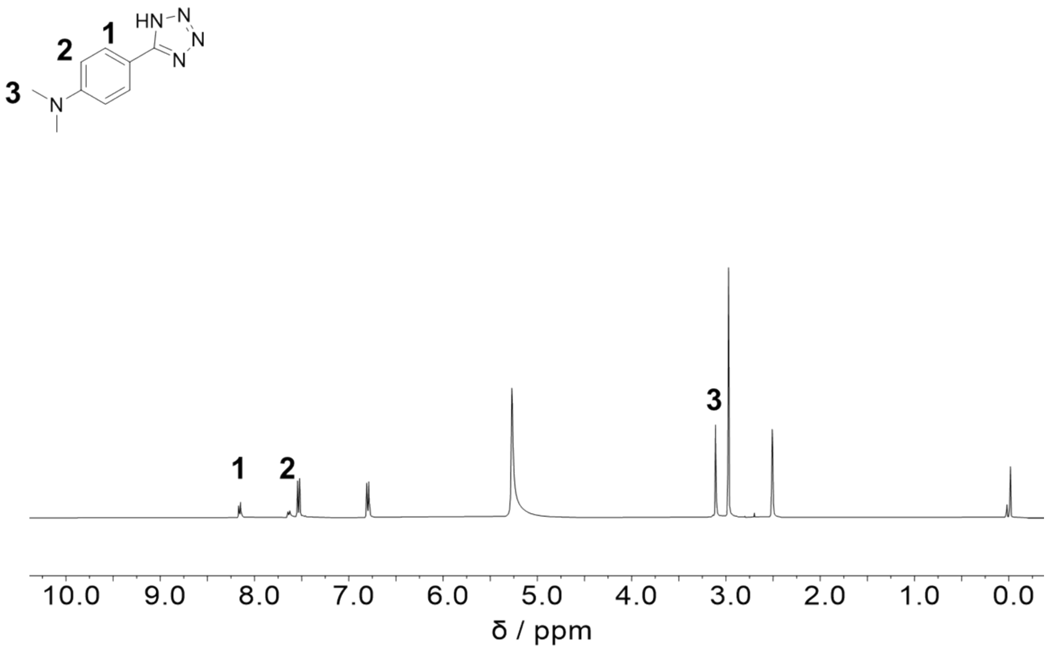

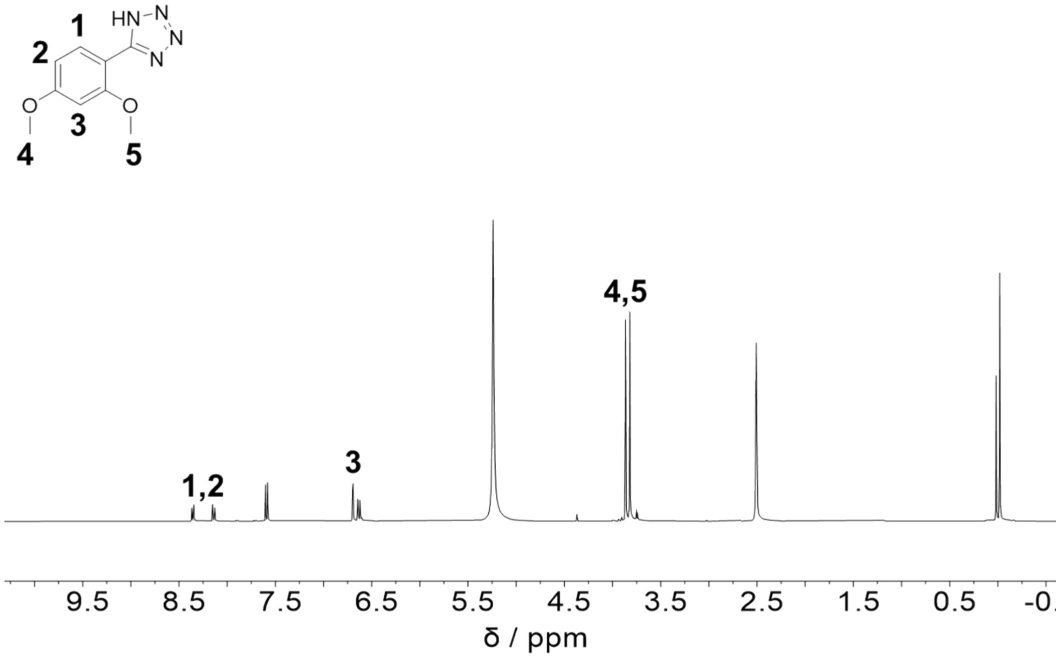

Figure S8 ^{1}H-NMR-spectrum of **3f** using polymer-bound $AlCl_3$ as catalyst in under bulk conditions at 160°C; spectrum was recorded in DMSO-d_6

ATR-IR Spectra

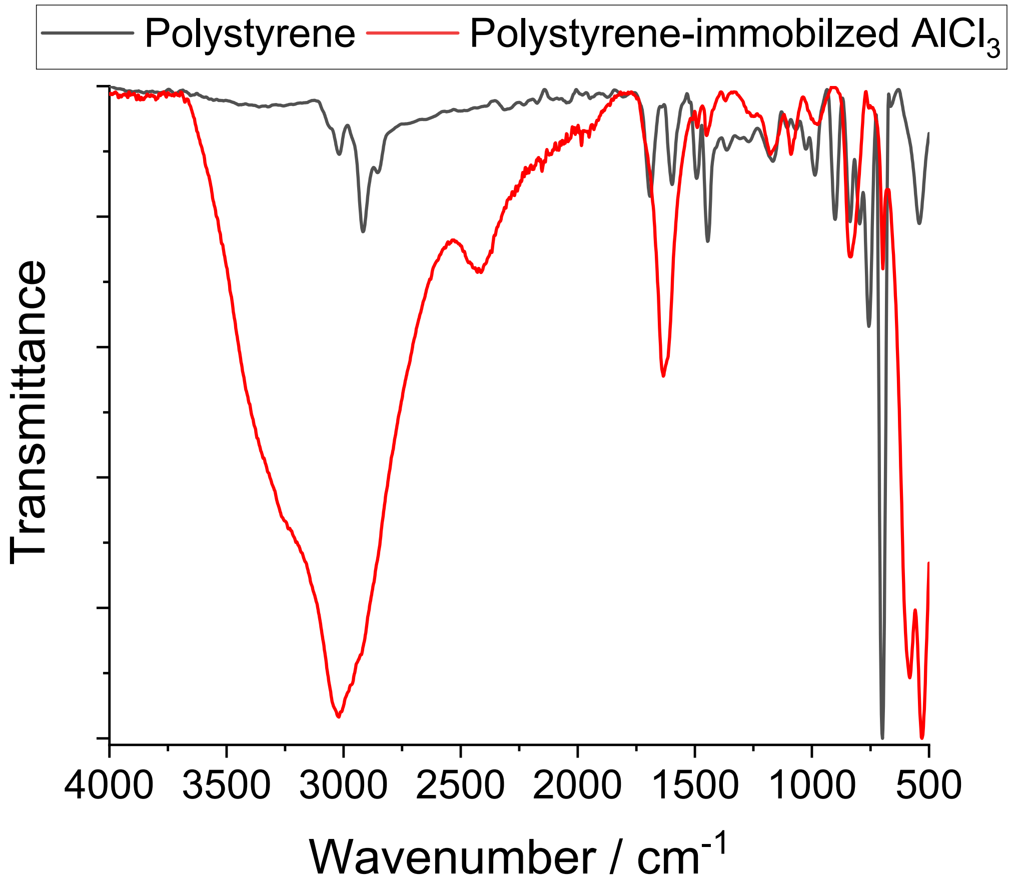

Figure S9. ART-IR spectra of polystyrene (black) and $AlCl_3$ immobilized in polystyrene (red)

Gas Sorption Measurements

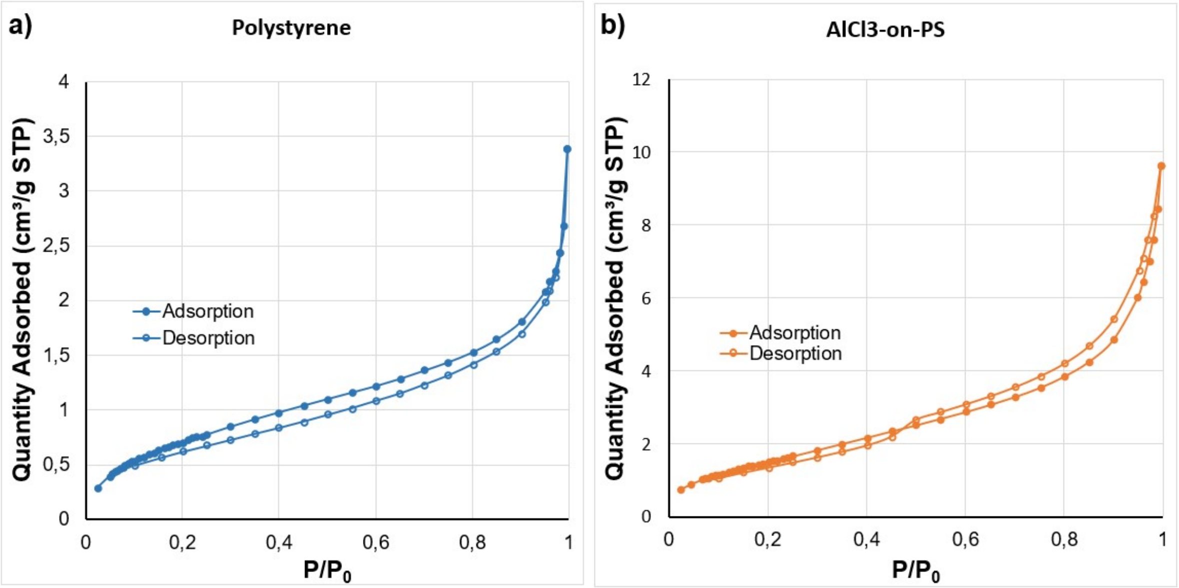

Figure S10. N2 adsorption isotherms. a) Polystyrene sample. b) AlCl3-on-PS

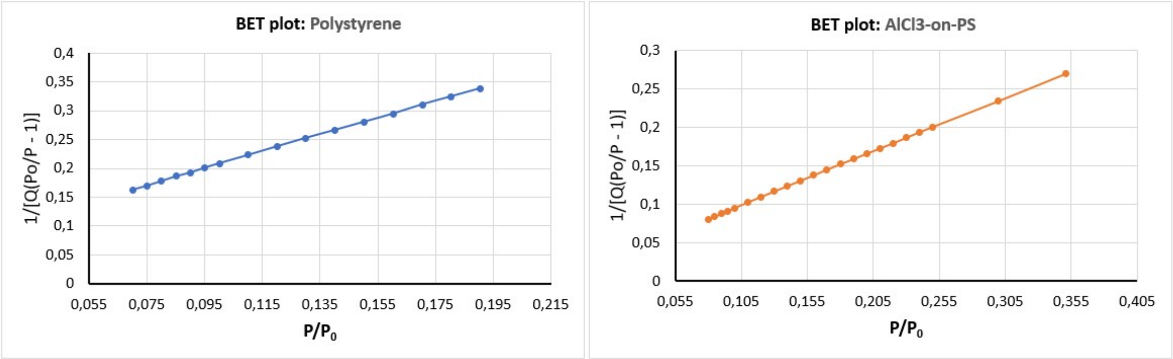

Figure S11. Brunauer-Emmett-Teller (BET) analysis

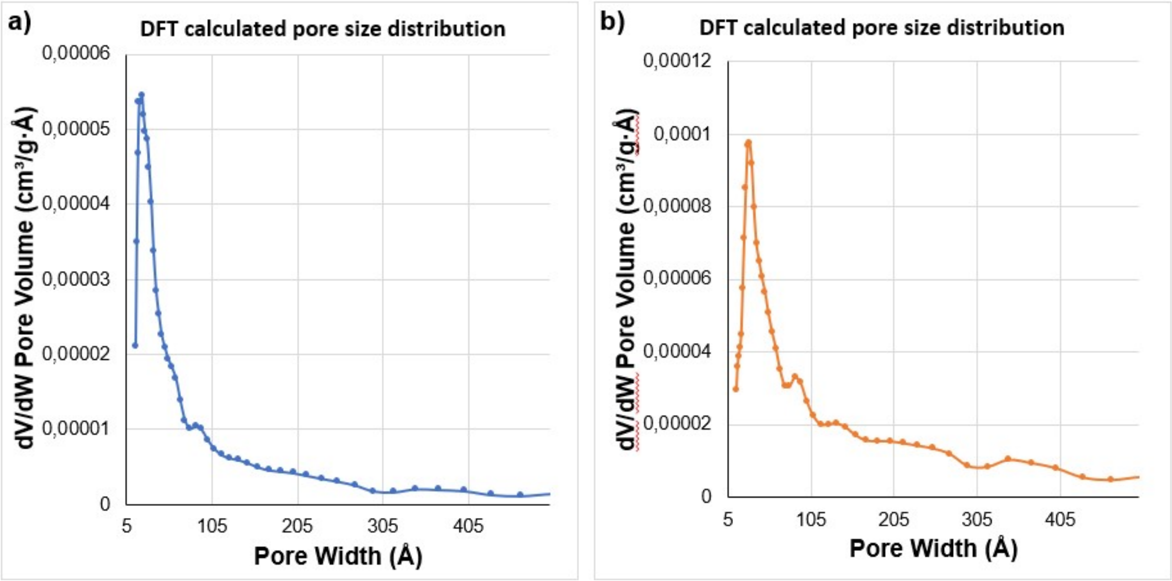

Figure S12. Pore size distribution analysis obtained from Density Functional Theory (DFT)

X-Ray Powder Diffraction Patterns

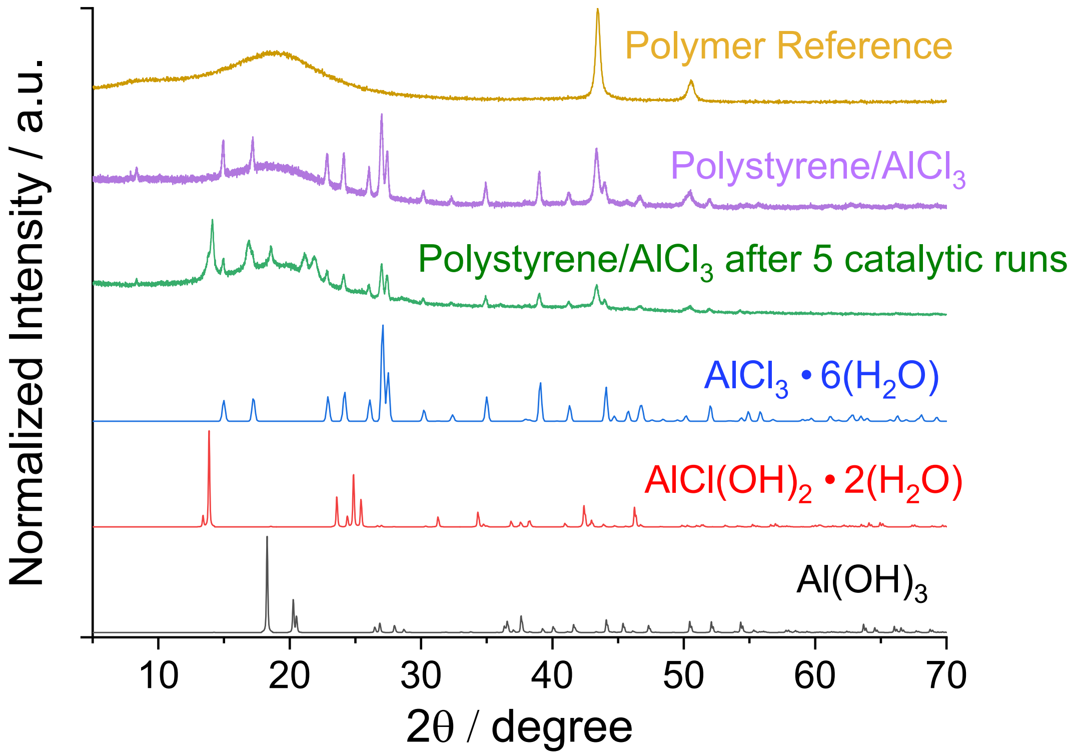

Figure S13. Normalized PXRD patterns of the polymer reference without $AlCl_3$ (yellow), the polymer-bound $AlCl_3$ composite before (violet) and after 5 catalytic runs (green). The diffractograms of $AlCl_3$ • 6(H_2O) (ICSD-22071, blue), $AlCl(OH)_2$ • 2(H_2O) (ICSD-425880, red) and $Al(OH)_3$ (ICSD-6162, black) are shown for comparison.

Optimization of $AlCl_3$ concentration

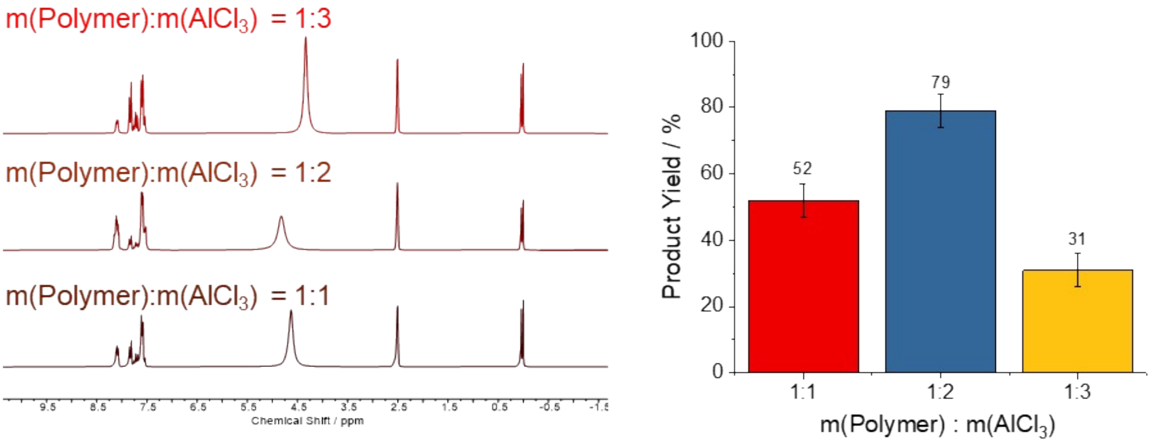

Figure S14 ^{1}H-NMR-spectra of **3a** using polymer-bound $AlCl_3$ prepared with different polymer-to-AlCl3 mass ratios as catalyst in under bulk conditions at 160°C; spectrum was recorded in DMSO-d6 (left) and comparison of the obtained product yields (right) rationalizing the 1:2 polymer:$AlCl_3$ ratio used in all further experiments
